# Classification of COPD patients and compliance to recommended treatment in Greece according to GOLD 2017 report: the RELICO study

**DOI:** 10.1186/s12890-021-01576-6

**Published:** 2021-07-09

**Authors:** Nikolaos Tzanakis, Nikolaos Koulouris, Katerina Dimakou, Konstantinos Gourgoulianis, Epameinondas Kosmas, Georgia Chasapidou, Athanasios Konstantinidis, Christos Kyriakopoulos, Theodoros Kontakiotis, Aggeliki Rapti, Mina Gaga, Konstantinos Kalafatakis, Konstantinos Kostikas

**Affiliations:** 1 Department of Respiratory Medicine, University General Hospital of Heraklion, Heraklion, Greece; 2 1st Department of Respiratory Medicine and Intensive Care Unit, Medical School, National and Kapodistrian University of Athens, Athens, Greece; 3 5th Respiratory Medicine Department, General Hospital for Chest Diseases “SOTIRIA”, Athens, Greece; 4 Department of Respiratory Medicine, Faculty of Medicine, University of Thessaly, BIOPOLIS, 41500 Larissa, Greece; 5 Department of Pulmonary Medicine PNOH, Metropolitan Hospital, Neo Faliro, Greece; 6 Department of Pulmonary Medicine, General Hospital of Thessaloniki “Georgios Papanikolaou”, Thessaloniki, Greece; 7 Respiratory Medicine Department, University Hospital of Ioannina, Ioannina, Greece; 8 2nd Respiratory Medicine Department, General Hospital for Chest Diseases “SOTIRIA”, Athens, Greece; 9 7th Respiratory Medicine Department, General Hospital for Chest Diseases “SOTIRIA”, Athens, Greece; 10 Department of Informatics and Telecommunications, School of Informatics and Telecommunications, University of Ioannina, Arta, Greece

**Keywords:** COPD, GOLD 2017 recommendations, ABCD assessment tool, Chronic management

## Abstract

**Background:**

Chronic obstructive pulmonary disease (COPD) is a multifactorial clinical condition, characterized by chronic progressive (or worsening) respiratory symptoms, structural pulmonary abnormalities, and impaired lung function, and is often accompanied by multiple, clinically significant comorbid disorders. In 2017, the Global Initiative for Chronic Obstructive Lung Disease (GOLD) issued a new report on COPD prevention, diagnosis and management, aiming at personalizing the maintenance therapeutic approach of the stable disease, based on the patients’ symptoms and history of exacerbations (ABCD assessment approach). Our objective was to evaluate the implementation of GOLD suggestions in everyday clinical practice in Greece.

**Methods:**

This was a cross-sectional observational study. Sixty-five different variables (demographics, vital sign measurements, COPD-related medical history parameters, comorbidities, vaccination data, COPD severity based on spirometry measurements, COPD stage based on the ABCD assessment approach, COPD treatments) were collected from 3615 nation-wide COPD patients (Greece).

**Results:**

The mean age at the time of initial COPD diagnosis was 63.8 (± 10.2). Almost 60% of the subjects were classified into group B, while the remaining patients were falling into groups A (18%) and D (21%), and only a small minority of patients belonged to Group C, according to the ABCD assessment approach. The compliance of respiratory physicians to the GOLD 2017 therapeutic suggestions is problematic, especially when it comes to COPD patients belonging to Group A.

**Conclusion:**

Our data provide valuable information regarding the demographic and medical profile of COPD patients in Greece, the domains which the revised ABCD assessment approach may show some clinical significance on, and the necessity for medical practitioners dealing with COPD patients to adhere closer to international recommendations for the proper management of the disease.

**Supplementary Information:**

The online version contains supplementary material available at 10.1186/s12890-021-01576-6.

## Background

Chronic obstructive pulmonary disease (COPD) is a multifactorial clinical condition, mainly related to smoking, characterized by chronic respiratory symptoms, structural pulmonary abnormalities, impaired lung function, or a combination of these findings, and is often accompanied by multiple, clinically significant comorbidities. According to current data on pathogenesis, COPD derives from various lifelong, dynamic, and cumulative interactions between genomic and environmental factors, that modulate the development, maintenance, and function of the lungs, through various biological mechanisms, including but not limited to airways’ and systemic inflammation [1]. It is this diversity in the etiopathogenesis, pathophysiology and clinical manifestations that makes early, accurate diagnosis and management of COPD particularly challenging. Possibly this is also the reason for the notable variance in the reported prevalence of COPD throughout the world [2], as researchers use different sampling schemes, diagnostic criteria, and measurement modalities. Various sources of data are currently available on the epidemiological features of COPD, either for USA [3], China [4], Europe [5] and worldwide [6].

In 2017 GOLD (Global Initiative for Chronic Obstructive Lung Disease) issued a new report for COPD diagnosis, management, and prevention. The goal of the GOLD assessment is to guide therapy based on the severity of airflow limitation as estimated by spirometry, the intensity of symptoms and the risk of future exacerbations or hospitalizations. Earlier GOLD guidelines [7] used a simple spirometric grading system to classify COPD patients and determine optimal treatment. In 2011, this system was updated to also include the symptomatic assessment and the risk of exacerbation of each patient [8]. The so-called “ABCD” tool combines the data from spirometry, the modified British Medical Research Council questionnaire (mMRC) or the COPD Assessment Test (CAT) and the history of patient’s exacerbations to classify patients into four groups. It has been noted though, that this classification had some limitations. Firstly, the ABCD assessment tool performed no better than the spirometric grades for mortality prediction or other important health outcomes in COPD. Secondly, group “D” outcomes were modified by two parameters: lung function and/or exacerbation history, which caused confusion. Under this perspective, in 2017, a revision of the ABCD assessment tool was proposed that separated spirometric grades from the “ABCD” groups. Therefore, grading according to spirometry as well as group classification according to patient-reported symptoms (CAT and mMRC assessment) and exacerbations were reported for each patient [9].

A previous study in Greece has demonstrated that the overall prevalence rate in the studied population (adults 35 years old and smokers of at least 100 cigarettes per lifetime) was 8.4%. It was found that 11.5% of the men and 4.8% of the women had spirometrically confirmed COPD [10]. Another two, more recent studies, with much narrower geographical coverage, estimated a COPD prevalence of 9% (mean patients’ age 52 years) in parts of northern Greece [11] and 18% (mean patients’ age 54 years) in Thessaly region [12]. However, a substantial number of COPD patients remain undiagnosed. In addition, there is little information regarding the treatment of COPD patients in the real world setting and the adherence to the GOLD recommendations. We have hypothesized that these changes in GOLD recommendations were not yet fully integrated in clinical practice in Greece. In any case, few data exist for the real-world treatment of COPD in Greece and even fewer for the adherence of physicians to treatment recommendations. The aim of the present study was to investigate (1) the everyday clinical practice on the COPD therapeutic management in Greece, (2) the severity of the disease in relation to the treatment provided and (3) the correlation of the data with GOLD 2017 recommendations.

## Materials and methods

### Bioethical considerations

The study was conducted according to the provisions of Good Clinical Practice, local laws, EU-Directive 2001/20, and the International Conference on Harmonization and the World Medical Association Declaration of Helsinki guidelines. Prior its initiation, study protocol and all relevant documentation was submitted to and approved by the Institutional Review Board/ Ethics Committees of the following institutions: “Agios Savvas” General Oncology Hospital of Athens, “Sotiria” General Hospital of Thorax Diseases of Athens, “Metropolitan” Hospital of Athens, “Attikon” University General Hospital of Athens, University General Hospital of Heraklion, “Evangelismos” University General Hospital of Athens, “G. Papanikolaou” General Hospital of Thessaloniki, “Euromedica” Hospital of Thessaloniki, University General Hospital of Ioannina and University General Hospital of Larisa. Given the sensitive nature of data processed in the frame of the study, all parties involved undertook adequate safety measures (physical, logical, organisational, technical, etc.) to warrant that data would always be processed safely and in compliance with the EU Data Privacy Directive 95/46/EC.

### Participants

Patients needed to fulfil all of the following criteria for being included into the study: (1) have an age ≥ 40 years, (2) established or new diagnosis of COPD, (3) do not undergo any exacerbations treatment neither at baseline visit nor in the previous month (i.e., have a stable COPD), (4) be able and willing to give informed consent and comply with study procedures. Male and female patients were recruited. Patients were excluded from the study if: (1) undergoing pregnancy or lactation (in case of female subjects), (2) they had a previous diagnosis of asthma, sleep apnea syndrome, other chronic respiratory disease other than COPD, or were classified as asthma-COPD overlapping patients (3) they had any acute or chronic condition that would limit the patient’s ability to complete questionnaires or participate in the study, or (4) they were participating in another study. Subjects were able to withdraw from the study at any time without explanation, without losing the right to medical care.

### Study objectives

The primary objectives of the study were to (1) record the stage of COPD patients according to GOLD 2017 and (2) record the treatment of COPD patients in relation to COPD stage in Greece and to correlate the data with GOLD 2017 suggestions. Secondary objectives of the study were to (3) describe the patients’ characteristics in relation to GOLD categories, (4) determine the comorbidities of the patients, treated or not, (5) record COPD exacerbations during the last year, (6) record information related to vaccination habits of that population, and (7) evaluate the compliance to the recommended treatment.

### Study structure and workflow

This was a cross-sectional observational study. Fifteen hospitals and 340 private practice respiratory physicians collaborated for this project across the Greek state; within a period of 5 months (March–August 2017), each investigator was scheduled to record sequentially 10 COPD patients, and each healthcare site was scheduled to record sequentially 20 COPD patients. The predefined standardised workflow of the study included the following steps: (1) recruitment of sequentially presenting patients with COPD diagnosed by a pulmonologist and stable disease at the time of enrollment (see inclusion criteria), (2) signing and dating of the informed consent form by the patient, (3) implementation of the complete initial medical examination and completion of the rest of the standardised case report form of the study, including (4) documentation of compliance to the treatment for COPD, by reporting how many doses they miss on average on a weekly basis (independent of the current study, which was not an interventional study), (5) end of recruitment, (6) data anonymisation, storing and locking, (7) data processing and statistical analysis respecting data integrity and safety (Additional file 1: Figure S1).

### Outcome measures

*Demographics* participants were registering their age (in years), gender (male, female), height (in cm) and weight (in kg).

*Vital sign measurements* investigators were recording the diastolic and systolic blood pressure of the subjects (in mmHg) as well as their heart rate (beats per minute) and respiratory rate (breaths per minute.)

*Medical history related to COPD* participants were asked to provide the following pieces of information: (1) date of initial COPD diagnosis (otherwise unknown), (2) current COPD treatment (multiple choice among thirteen categories; short-acting β adrenoreceptor agonists/SABA, long-acting β adrenoreceptor agonists/LABA, short-acting muscarinic receptor antagonists/SAMA, long-acting muscarinic receptor antagonists/LAMA, methylxanthines, inhaled corticosteroids/ICS, oral glucocorticoids, phosphodiesterase-4 (PDE4) inhibitors, antibiotics, antioxidants, mucolytic agents, combination of LAMA + LABA, combination of LABA + ICS), date of initiation and frequency of missing doses (never, once monthly, twice monthly, once weekly, twice weekly) per pharmacological category, (3) number of COPD exacerbations during the last 12 months and number of them having led to the emergency department or having required hospitalization.

*Comorbidities* participants were asked to provide information on any comorbidities, indicate the date of diagnosis and whether they receive any concomitant medication.

*Vaccination data* participants were asked to confirm whether they were vaccinated for influenza (during the running year) and/or pneumococcus.

*Spirometric assessment of pulmonary function* available data of the most recent pre- and post-bronchodilation spirometry according to common clinical practice were utilised [forced expiratory volume in 1s (FEV_1_) (in L and % predicted) and the FEV_1_ to forced vital capacity (FEV_1_/FVC) ratio]. No date-limit was set; 60.8% of subjects underwent spirometry on the date of recruitment, 2.8% underwent spirometry after that date, 33.8% provided data up to 1 year old, and 2.6% provided data older than that. Finally, subjects were kindly requested to complete the CAT. CAT consists of 8 items, each of which describes the best to worst case of a state on a 0–5 scale (for instance, for the state “coughing” the best case is “I never cough”, corresponding to 0, and the worst case is “I cough all the time”, corresponding to 5. Values 2–4 represent intermediate cases between the two extremes). CAT score comprises the sum of scale values for all 8 items, thus ranges from 0 to 40.

### Statistical analysis

After checking the collected data for completeness, they were double entered into Epi-data version 3.1 and exported into SPSS version 21 for analysis. In this study sample, any missing values were not replaced by others and the calculations were based on the number of cases for which there is information. The data were processed by using descriptive analysis, including frequency distribution, cross tabulation, and summary measures.

For the comparison of quantitative characteristics with normal distribution of values between the different groups that will be compared, t-test was used for independent test observations pairs or analysis of variance, if comparisons took place between more than 3 groups or multiple categorical factors existed. Two-tailed tests were performed for all analyses, and *P* was set to 0.05. All results shown in the corresponding tables and figures are mean ± standard deviation (SD). Bonferroni correction has been applied to account for multiple comparisons.

## Results

*Data acquisition* 3740 subjects were initially screened and 125 of them excluded from the study, because they did not cover all inclusion criteria and/or at least one exclusion criterion was applicable. After recruitment, no discontinuation of subjects has been confirmed. Thus, datasets from 3615 COPD patients were collected. Various missing values have been retrospectively (at the stage of data processing) encountered (Additional file 1: Figure S1).

*Demographics* male to female ratio was 3:1 in our study sample (male participants 73.9%) with an age ranging from 40 to 95 years (mean and median age 69 years, with a SD of 10 years). Two thirds of our study sample belonged to the 60–79 years age group (67.7%), while the remaining third was roughly divided between the 40–59 years (18.5%) and the 80–99 years age group (13.8%). The mean (and median) body mass index (BMI) was higher than 27 (with a SD of 5.1) with almost three quarters of our study sample belonging to the overweight (25–29.9, 43%) or obese (30 + , 29.2%) BMI group. Only forty-eight subjects (1.3%) were underweighted to severely underweighted (Additional file 1: Figure S2). It should be noted that two subjects < 40 years of age were mistakenly classified as eligible study participants, and their data were included into the analysis. Nevertheless, they represent only 0.05% of the study sample, therefore not altering the results presented or the study inferences.

*Vital signs* The mean systolic/diastolic blood pressure (± SD) was 130/79 (12.5/9.4), the mean heart rate (± SD) 80 (9.6) and the mean respiratory rate (± SD) 18.2 (9.3). No differences were found in BMI, systolic/ diastolic blood pressure, heart, and respiratory rates between the 3 age groups (40–59, 60–79 and 80–99 years) among men and women (Additional file 1: Figure S2).

**Fig. 1:**
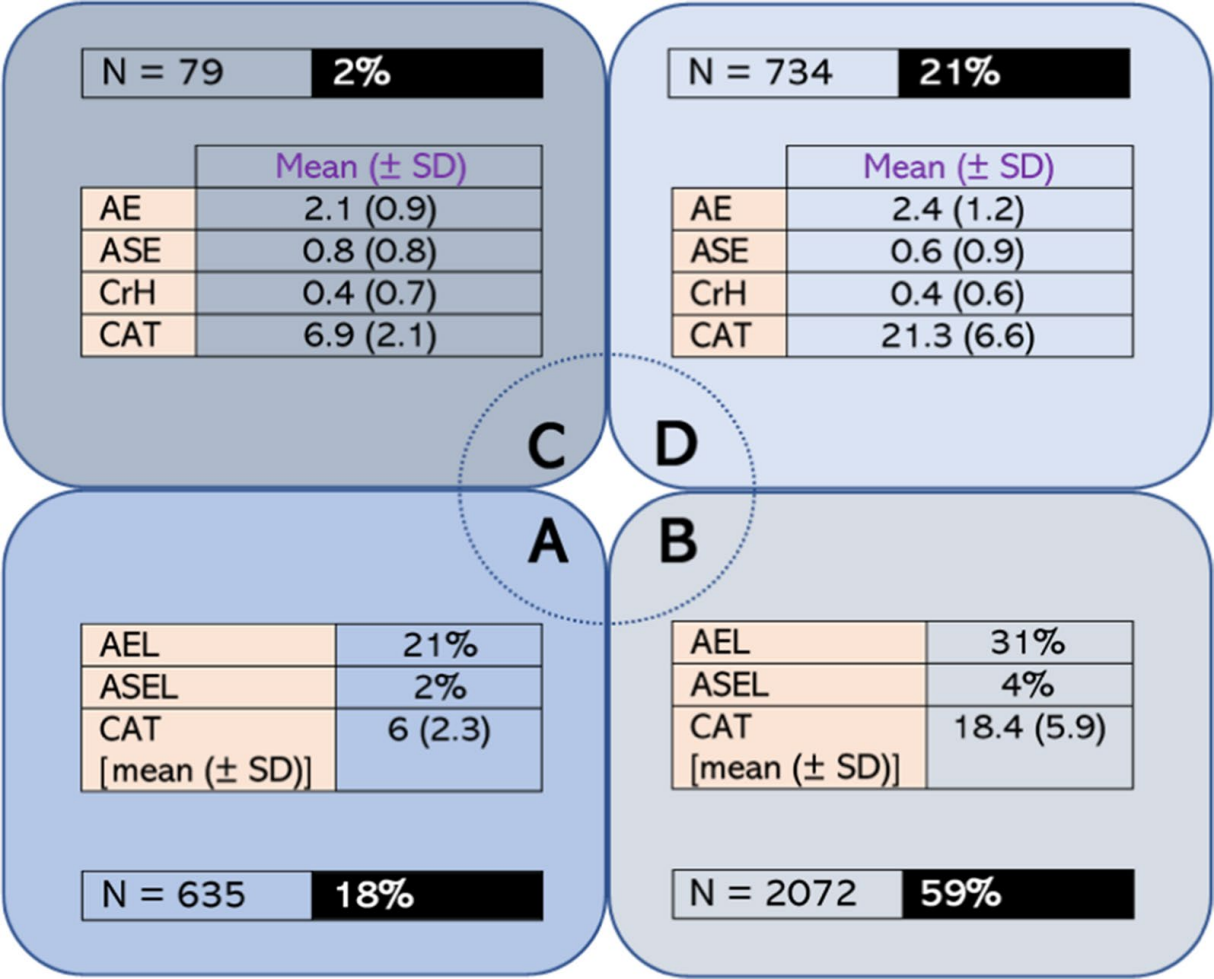
Classification of COPD patients to one of four groups, based on the ABCD classification tool (N = 3520), which takes into consideration the overall score in the CAT as well as the patients’ history of exacerbations, mild or leading to hospitalization, over the last 12 months. AE: annual exacerbations, ASE: annual severe (i.e. leading to the emergency department) exacerbations, AEL: annual exacerbation likelihood, ASEL: annual severe (i.e. leading to the emergency department) exacerbation likelihood, CAT: COPD assessment test, COPD: chronic obstructive pulmonary disease, CrH: COPD-related hospitalizations

*COPD-related medical history* we calculated the time since initial COPD diagnosis and subtracted it from the current age of participants (i.e. age at recruitment) to estimate their age at first COPD diagnosis. The available data (N = 2552) indicate that the mean age (± SD) at COPD diagnosis was 63.8 years (10.2) and that 85% of patients were between 50–79 years of age at the time of diagnosis (Additional file 1: Figure S2). According to the subjects’ overall CAT score (Additional file 1: Table S1), their history of COPD exacerbations, either mild or serious enough to lead to hospitalization (Additional file 1: Table S2), participants were classified into one of four groups of the ABCD assessment tool. Based on the available data (N = 3520) more than half (59%) of the subjects were classified as belonging to group B, while almost all of the remaining cases were classified as belonging to groups A (18%) and D (21%). Only a tiny portion of the participants (2%) was categorized into group C (Fig. 1).

**Fig. 2:**
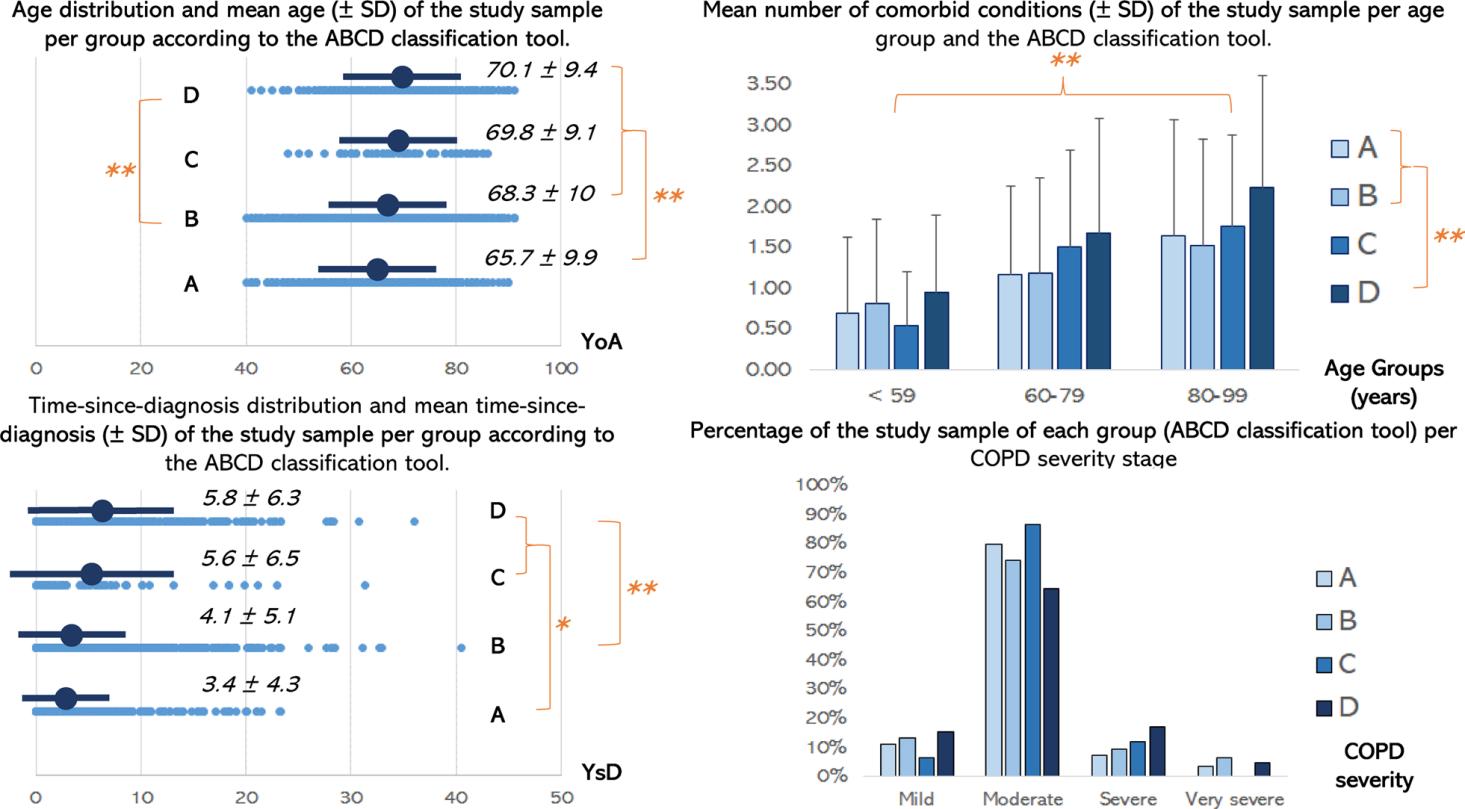
(Upper left panel) Relationship between the ABCD classification method with the age of COPD patients. (Lower left panel) Relationship between the ABCD classification method with the time passed since the initial diagnosis of COPD patients. (Upper right panel) Relationship between the ABCD classification method with the number of other, comorbid conditions of COPD patients. (Lower right panel) Relationship between the ABCD classification method with the severity of the disease of COPD patients. COPD: chronic obstructive pulmonary disease, SD: standard deviation, YoA: years of age, YsD: years since diagnosis. * *p* ≤ 0.01, ** *p* ≤ 0.00

*ABCD assessment tool in relation to age and years since diagnosis of patients* significant differences exist between the mean age [F_3,2484_ = 18.06, *p* < 0.001] or the time since initial COPD diagnosis of subjects (Additional file 1: Figure S3) [F_3,2484_ = 19.88, *p* < 0.001] belonging to each of the four groups based on the ABCD assessment tool. Overall, both variables tend to slightly increase when moving to a higher group. From the available data (N = 2485) we conclude that subjects in group A are on average 2.5 years younger compared to those belonging to group B (*p* < 0.001) and more than 4 years younger compared to those belonging to group C (*p* = 0.008) or D (*p* < 0.001). Moreover, subjects in group B are almost 2 years younger compared to those in group D (*p* = 0.002). Similarly, subjects in group A were diagnosed with COPD more recently, at least 6 months, compared to those in group B (*p* = 0.052) and at least 2 years from those in groups C (*p* = 0.01) or D (*p* < 0.001). The same applies for subjects in group B compared to those of group D (*p* < 0.001) (Fig. 2). No associations were found between ABCD group allocation with gender or BMI (Additional file 1: Table S3).

*ABCD assessment tool in relation to comorbid conditions of patients* the group of the ABCD assessment tool that the patients belong to and their age group (< 59, 60–79, 80–99 years) relate to the burden of comorbid conditions of COPD patients, according to the available data (N = 3518). In particular, subjects belonging to group D were much more likely to suffer from a second comorbid condition (excluding COPD) compared to subjects belonging to groups A or B, who were predominantly diagnosed with just one comorbid condition [F(3, 21.6) = 15.3, *p* < 0.001]. Furthermore, subjects belonging to a higher age group gradually increased their likelihood of having one or two comorbid conditions (excluding COPD) [F(2, 39.9) = 28.4, *p* < 0.001] (Fig. 2). A list of the comorbid conditions of this study sample can be found in Additional file 1: Table S4.

*ABCD assessment tool in relation to severity of airflow limitation* the severity of airflow limitation was determined based on each patient's latest available spirometric results (Additional file 1: Figure S4) as mild (FEV_1_ ≥ 80%pred), moderate (FEV_1_ = 50–79%pred), severe (FEV_1_ = 30–49%pred) and very severe (FEV_1_ < 30% %pred). Most subjects, independent of their ABCD group allocation, were measured as suffering from moderate COPD. Moreover, the portion of subjects suffering from severe COPD increased as we move from group A to group D (Fig. 2).

*ABCD assessment tool, COPD treatment practices and vaccination* combined therapy with LAMA/LABA bronchodilators was the most frequently prescribed therapeutic option in our study sample (applied to 48% of the subjects), while other frequent options included combination of LABA, LAMA and ICS (28% of subjects) (Additional file 1: Figure S5). As a short acting bronchodilator, SABA was the most frequently used option (15.5% of subjects) (Additional file 1: Table S5). By combining the available data from the ABCD classification tool and the treatment choices of the corresponding subjects (N = 3520), it appears that only 18% of group A patients were treated according to the GOLD 2017 suggestions, while the corresponding percentage for group B and C patients was 59% and 58%, respectively. Treatment of group D patients showed the highest percentage of adherence to GOLD 2017 recommendations (82%) (Additional file 1: Figure 3). Finally, subjects were showing higher probability for being vaccinated against both, Influenza virus and S. pneumoniae, or at least one of them, if suffering from severe or very severe COPD, or belonging to groups B and D (Additional file 1: Table S6). More specifically, over 82% of severe to very severe COPD patients (or patients belonging to groups B and D) were vaccinated against at least one of the pathogens, while this value drops to below 75% for patients with mild COPD (or those belonging to groups A and C).

**Fig. 3:**
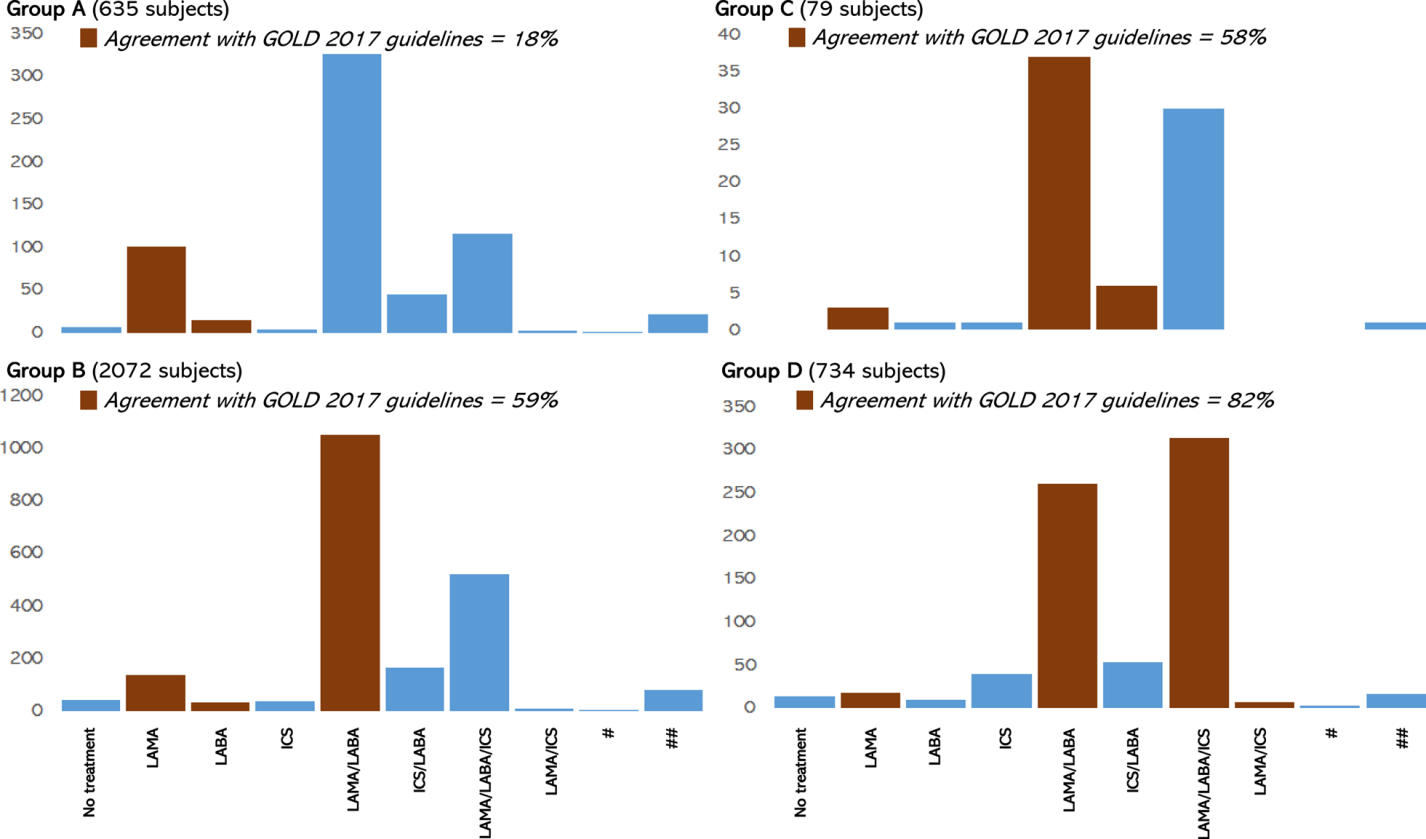
Different treatment strategies prescribed to the subjects per ABCD group, and registration of them to the GOLD 2017 guidelines. ICS: inhaled corticosteroids, LABA: long-acting β adrenoreceptor agonists, LAMA: long-acting muscarinic receptor antagonists, #: 2 bronchodilators of the same type plus ICS, ##: 3 or more bronchodilators

*Compliance to treatments* Perfect compliance for most drug categories (referring either to LAMA, LABA, ICS and their combinations, or to supporting treatments for symptomatic relief, such as short-acting bronchodilators or methylxanthines) was at the level of 65–75%, with the exception of antibiotics (this refers to prophylactic low dose azithromycin in a small number –110– of patients, approaching 85%), mucolytic agents and antioxidants (just above 50%). Poor compliance (missing doses with a frequency of more than twice weekly) was in most cases not exceeding 10%, and in the cases of the principal treatment options (LAMA, LABA, ICS and their combinations) not exceeding 6% (Additional file 1: Table S7, Figure S5).

## Discussion

This large COPD non-interventional, epidemiological study primarily focused on generating real-world data related to the course of COPD patients under typical treatment in the community [13, 14]. The short duration of the study and the enrollment of sequential patients from each participating physician and healthcare site aimed at avoiding the selection bias and include a representative population of Greek COPD patients. The study provided valuable information regarding the demographic and medical profile of COPD patients in Greece, their classification based on the revised ABCD assessment tool, and whether clinicians in Greece prescribe medications for the chronic treatment of the disease in agreement with the GOLD 2017 recommendations. It is worth mentioning that the main reason for designing this study around the GOLD classification and adherence to the 2017 revision is the lack of any national (Greek) guidelines published or proposed in COPD and the fact that most physicians of the country refer to the GOLD documents for the management of COPD.

The outcomes of this study indicate that only an exceedingly small fraction of COPD patients could be registered into group C of the ABCD assessment tool, proposed under the revised GOLD 2017 suggestions. Only a small minority of patients suffered from two or more annual exacerbations, or at least from one serious enough to lead to hospitalization, and at the same time claim that their daily routine is almost as good as normal (CAT score < 10). Moreover, this study shows that there was no strong relationship between the ABCD classification tool with COPD severity, as indicated by the spirometric measurements. Most patients, from all ABCD groups, suffer from COPD of moderate severity.

Moreover, the ABCD classification system corelates with the age of COPD patients, the duration of their disease, as well as the number of comorbid conditions they suffer from. Patients of Group C and D were on average older than those of Group B, who were on average older than those of Group A. Similarly, patients of Groups C and D suffered from COPD for a longer period compared to those of Group B, who have suffered from COPD for a longer period compared to those of Group A. Finally, Group D patients had significantly more chances of having three chronic conditions (including COPD) compared to Group A or B patients, who usually suffered from up to one more chronic condition (besides COPD).

Furthermore, the outcomes of this study indicate that, from a therapeutic point of view, there is an astonishingly low adherence to GOLD 2017 recommendations for clinicians in Greece, when it comes to Group A patients (just 18%), a moderate adherence when it comes to Group B or C patients (58–59%) and a good but not perfect adherence (exceeding 80%) when it comes to Group D patients. In patients belonging to Groups A–C, deviations from GOLD 2017 suggestions refer mainly to prescribing more drugs than necessary, or inappropriate drugs, rather than prescribing less drugs than supposed to. We should note, though, the fact that the ABCD tool is aimed at being used for the initial assessment and management of patients with COPD, and after receiving treatments, many patients experience a decrease in their symptoms and exacerbations frequency, thus if reassessed (like in the case of this study) with the ABCD tool, they may be grouped to less severe groups, only because their disease is well controlled by the treatments they receive. This could be an alternative explanation for this impressive mismatch between ABCD classification and applied treatment practices in Greece. If this is the case, COPD specialists need to consider more adequately de-escalation from initial pharmacological interventions. It should be noted that overtreatment of COPD patients in all GOLD groups, outside of evidence-based recommendations, is a common phenomenon observed both in primary care and in specialist settings [15].

The distribution of COPD patients into the different ABCD groups of the GOLD 2017 recommendations shows similarities as well as some differences, compared to other studies with similar recruitment criteria. For instance, only 2% of our study participants were distributed to Group C, which is the same portion also reported by Cui et al. [16] in China (in a sample of over 1200 patients), Tudoric et al. [17] from a sample of over 3300 patients from central and eastern Europe, and Duarte-de-Araújo et al. [18] in Portugal (in a sample of over 300 patients), although higher percentages have been also reported [19]. In the great majority of studies, just like in our work, group C is the smallest among the four groups of the ABCD system [16–18, 20–22], group B the biggest followed (in a few cases exceeded) by group D [16–22]. The sociodemographic profile of our study sample is also remarkably close to that reported by other similar studies. Finally, other studies -similarly to ours- report the best adherence to GOLD 2017 treatment guidelines for Group D and increasing overprescribing practices as we move to previous groups [18–21].

Two strong points of this study are the large number of COPD patients it contains and its nationwide extant, across the Greek territory. On the other hand, it is known that the GOLD report uses a fixed FEV1/FVC value (0.70) to define airway obstruction. This approach leads to a significant percentage of false-positive diagnoses of mild and moderate COPD. These patients, who are in good respiratory health may be incorrectly identified as having an abnormally low FEV1. This is a long-standing controversy with the use of the lower limit of normal for the FEV1/FVC ratio. Finally, as mentioned above, similar studies have been conducted in other parts of the world.

Furthermore, all patients included in the study were stable COPD patients, managed by specialist pulmonologists either in the outpatient clinics of the hospitals or in private practices. A part of the private practice visits may have been covered by the patients’ insurance and part on private expenses, whereas the vast majority of the hospital visits were covered by the patients' insurance. Since the study was not part of a referral program for COPD, we should not expect differences between the two populations. The analysis of all data was performed considering all the patients as a single population.

## Conclusions

Based on the demographics and COPD-related medical history data of the study sample, we could infer that the most commonly encountered COPD patient involves an overweight or obese male individual aged > 50 years, usually with one or two other chronic comorbid conditions (most likely some type of cardiovascular disease), having a CAT score > 10 (thus belonging to group B or D according to the ABCD classification tool) and spirometric parameters indicative of a moderate stage of the disease, who also suffers from up to two, usually mild, exacerbations annually. Our data show a significant discrepancy between the complex ABCD system of the GOLD 2017 classification and real-life clinical practice that may reflect both the complexity of that classification and the need for better dissemination of treatment recommendations to practicing clinicians.

## Supplementary Information

**Additional file 1**: Figure S1: Pipeline of the different stages of this cross-sectional observational study. Figure S2: Summary of the demographic features and vital sign measurements of the study sample. Table S1: Summary statistics on CAT questionnaire. Table S2: Summary of COPD exacerbations within the last 12 months. Figure S3: Classification of subjects based on months since initial COPD diagnosis. Table S3: Subject allocation based on gender or body mass index and ABCD assessment. Table S4: List of the comorbid conditions present in COPD patients. Figure S4: COPD severity (stage) based on FEV1 preBD% of predicted. Figure S5: Colourmaps depicting the combination of principal treatments used by the study participants per group of the ABCD assessment tool, and their compliance to each of them. Table S5: Use of other pharmacological agents, for symptomatic treatment, besides the main treatment options for the chronic management of COPD (LAMA, LABA, ICS). Table S6: Distribution of vaccinated patients in ABCD groups. Table S7: Compliance of the study sample to the different COPD-related treatment categories.

## Data Availability

All data of the study have been presented in this manuscript and the corresponding supplementary materials.

## Abbreviations

BMI: Body-mass index
CAT: COPD assessment test
COPD: Chronic obstructive pulmonary disease
FEV_1_: Forced expiratory volume in 1s
FVC: Forced vital capacity
GOLD: Global Initiative for Chronic Obstructive Lung Disease
ICS: Inhaled corticosteroids
LABA: Long-acting β adrenoreceptor agonists
LAMA: Long-acting muscarinic receptor antagonists
mMRC: Modified British Medical Research Council questionnaire
PDE4: Phosphodiesterase-4
SABA: Short-acting β adrenoreceptor agonists
SAMA: Short-acting muscarinic receptor antagonists
