# Supplementary material for: Classification of COPD patients and compliance to recommended treatment in Greece according to GOLD 2017 report: the RELICO study
Source: BMC Pulm Med. 2021 Jul 9;21:216. doi: 10.1186/s12890-021-01576-6 (PMC8268415; doi:10.1186/s12890-021-01576-6)
Supplement: Supplementary file 1 — Additional file 1: Figure S1: Pipeline of the different stages of this cross-sectional observational study. Figure S2: Summary of the demographic features and vital sign measurements of the study sample. Table S1: Summary statistics on CAT questionnaire. Table S2: Summary of COPD exacerbations within the last 12 months. Figure S3: Classification of subjects based on months since initial COPD diagnosis. Table S3: Subject allocation based on gender or body mass index and ABCD assessment. Table S4: List of the comorbid conditions present in COPD patients. Figure S4: COPD severity (stage) based on FEV1 preBD% of predicted. Figure S5: Colourmaps depicting the combination of principal treatments used by the study participants per group of the ABCD assessment tool, and their compliance to each of them. Table S5: Use of other pharmacological agents, for symptomatic treatment, besides the main treatment options for the chronic management of COPD (LAMA, LABA, ICS). Table S6: Distribution of vaccinated patients in ABCD groups. Table S7: Compliance of the study sample to the different COPD-related treatment categories. [file 12890_2021_1576_MOESM1_ESM.pdf]

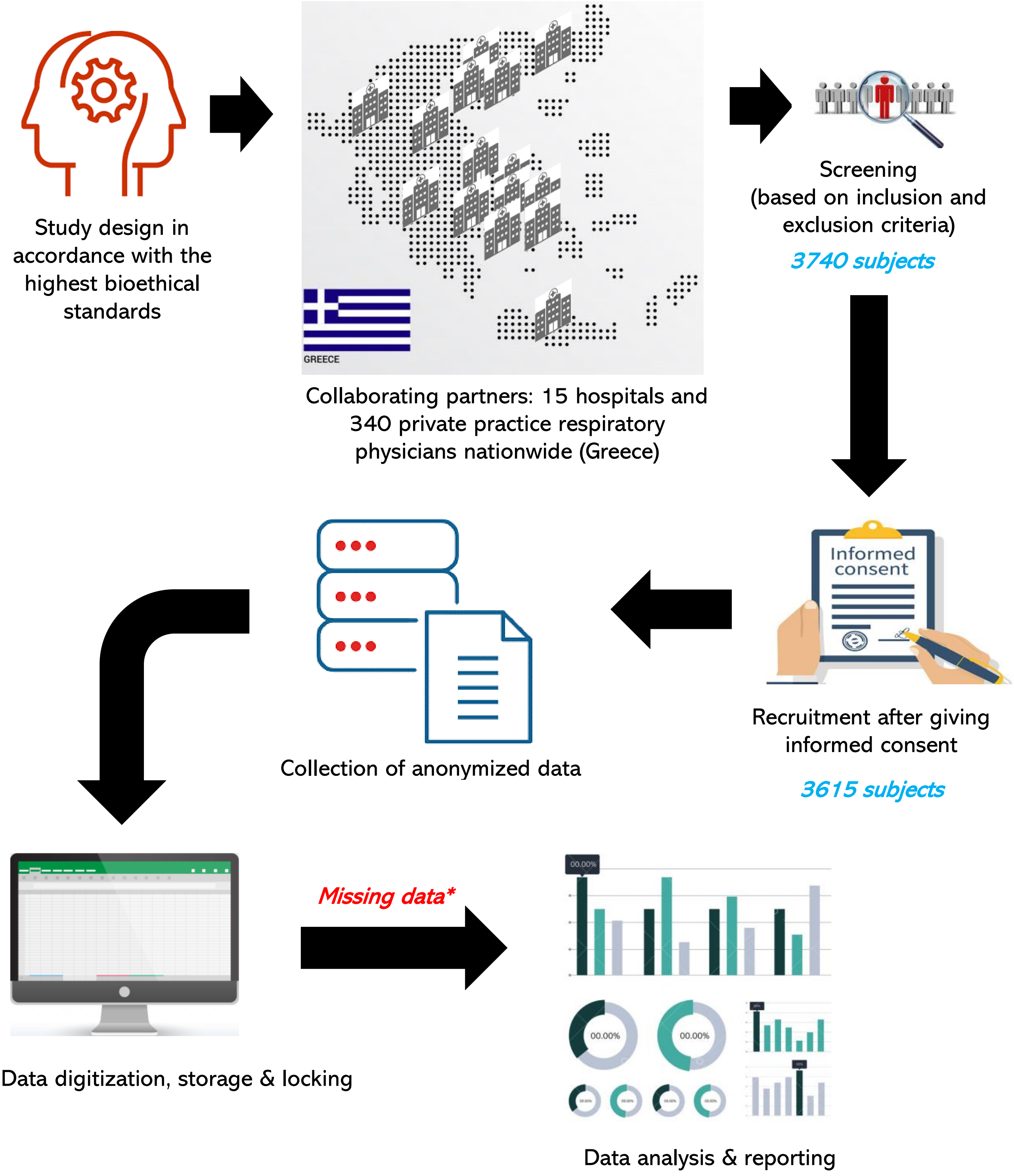

Supplementary Figure 1

Pipeline of the different stages of this cross-sectional observational study. The finalized database contains data from 3615 subjects. Sixty-five different parameters (variables) were collected per subject. Various isolated missing values have been retrospectively identified in some of the subjects (*). These relate to either the subjects' or the medical doctors' negligence to complete all domains of the corresponding case report form, which occurred in a random manner i.e., cannot introduce a systematic bias into the study.

CAT: COPD assessment test, COPD: chronic obstructive pulmonary disease

**Gender (N=3604)**	
Male	2665 (73.9%)
Female	939 (26.1%)
**Age (years) (N=3613)**	
VALUE RANGE	32-95
MEDIAN	69
MEAN (+/- SD)	68.6 (10)
**Age group (N=3613)**	
< 40 YoA	2 (0.05%)
40-59 YoA	668 (18.5%)
60-79 YoA	2444 (67.65%)
80-99 YoA	499 (13.8%)
**Height (cm) (N=3615)**	
VALUE RANGE	138-205
MEDIAN	170
MEAN (+/- SD)	169.2 (8.5)
**Weight (kg) (N=3614)**	
VALUE RANGE	40-197
MEDIAN	79
MEAN (+/- SD)	80 (16.6)
**BMI (kg/m^2) (N=3614)**	
VALUE RANGE	14.7-72.4
MEDIAN	27.4
MEAN (+/- SD)	27.9 (5.1)
**BMI group (N=3614)**	
< 18.5 kg/m^2	48 (1.3%)
18.5 – 24.9 kg/m^2	957 (26.5%)
25 – 29.9 kg/m^2	1554 (43%)
> 30 kg/m^2	1055 (29.2%)

		MEAN (+/- SD)			
		BMI	SBP / DBP	HR	RR
**MALE**	**40-59 YoA [N=450]**	28.8 (5.2)	130 (11.7) / 80 (9)	78.6 (9)	18.6 (11.3)
	**60-79 YoA [N=1811]**	27.9 (4.7)	130.7 (12.2) / 79.4 (9.3)	76.8 (9.5)	18 (9)
	**80-99 YoA [N=401]**	27.7 (4.3)	132.5 (13.5) / 79.8 (9.2)	75.9 (10.2)	17.9 (7.7)
**FEMALE**	**40-59 YoA [N=217]**	26.9 (6.5)	126.2 (12.1) / 78 (8.4)	77.1 (9.1)	19.3 (11)
	**60-79 YoA [N=626]**	27.7 (5.7)	128.5 (13) / 78.2 (9.6)	77.4 (8.8)	18.1 (9.2)
	**80-99 YoA [N=95]**	26.9 (4.2)	130.7 (14.6) / 79.8 (9.1)	76.4 (11.8)	19 (9.1)

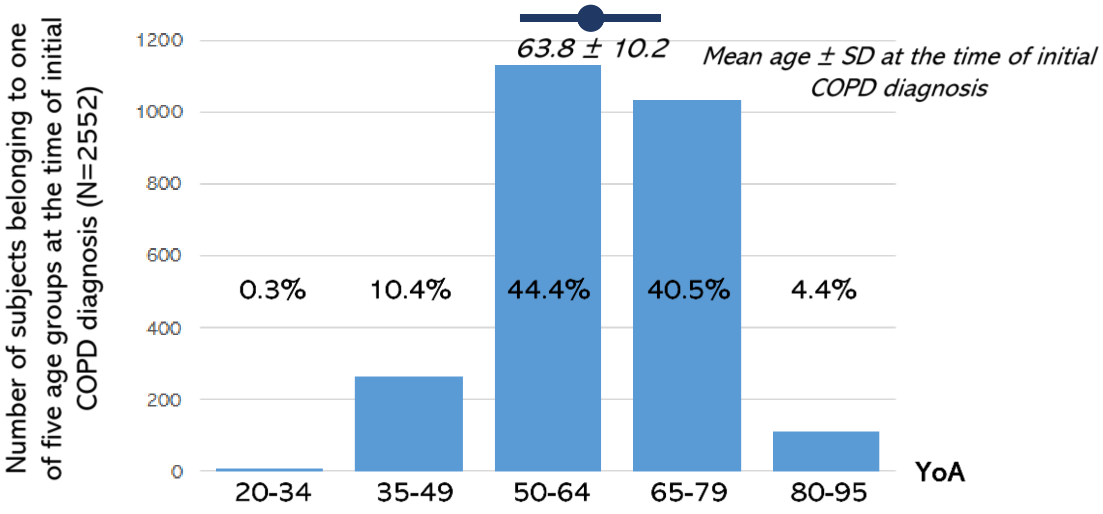

Supplementary Figure 2

(Left panel) Demographic characterization of the study sample. (Upper right panel) Summary statistics on the vital signs of the study sample divided by gender and age group. (Lower right panel) Distribution of age for the study sample at the time of initial COPD diagnosis. The great majority of individuals belonged to either the 50-64 YoA or the 65-79 YoA groups at the time of their initial COPD diagnosis. It should be noted that two subjects < 40 years of age were mistakenly classified as eligible study participants and were retained in data analysis, without thought affecting the study results or inferences.

BMI: body mass index (kg/m2), (S/D)BP: (systolic/diastolic) blood pressure (systolic/diastolic, in mmHg), HP: heart rate (min-1), RR: respiratory rate (min-1), SD: standard deviation, YoA: years of age

Supplementary Table 1

Summary statistics on CAT questionnaire

	N	MEAN	SD
**TOTAL SCORE (sum of partial domains)**	**3522**	**16.5**	**7.7**
Coughing	3610	2.2	1.1
Phlegm in the chest	3605	2.1	1.1
Feeling of tightness in the chest	3604	1.8	1.2
Breathlessness when walking up a hill or one flight of stairs	3612	2.9	1.3
Limitation in doing activities at home	3594	2.0	1.3
Confidence on leaving home despite the lung condition	3600	1.9	1.4
Sleeping soundly	3607	1.6	1.2
Having lots of energy	3592	2.2	1.2

CAT consists of 8 items, each of which describes the best to worst case of a state on a 0-5 scale.

N: number of values, SD: standard deviation

Supplementary Table 2

Summary of COPD exacerbations within the last 12 months

	N
Total number of COPD exacerbations in the study sample	2757
Total number of COPD exacerbations having led to the ER	602 (21.8%)
Total number of COPD exacerbations laving led to hospitalization	301 (10.9%)

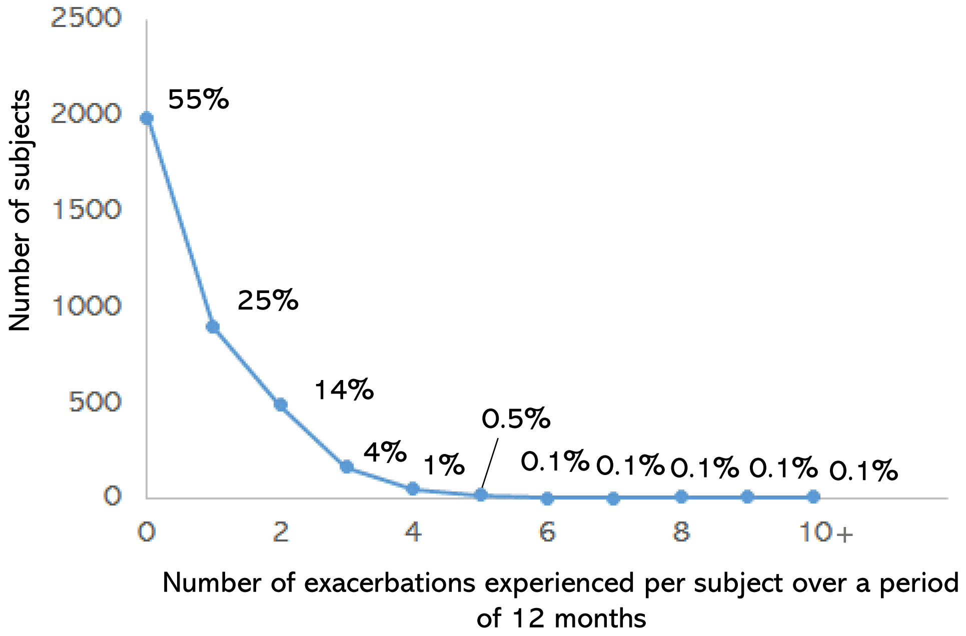

The great majority (94%) of the subjects who participated in this observational study had experienced maximum 2 exacerbations of COPD over the period of the last 12 months, usually no exacerbation (55%) or just one exacerbation (25%).

COPD: chronic obstructive pulmonary disease, ER: emergency room, N: number of cases

Supplementary Figure 3

Classification of subjects based on months since initial COPD diagnosis

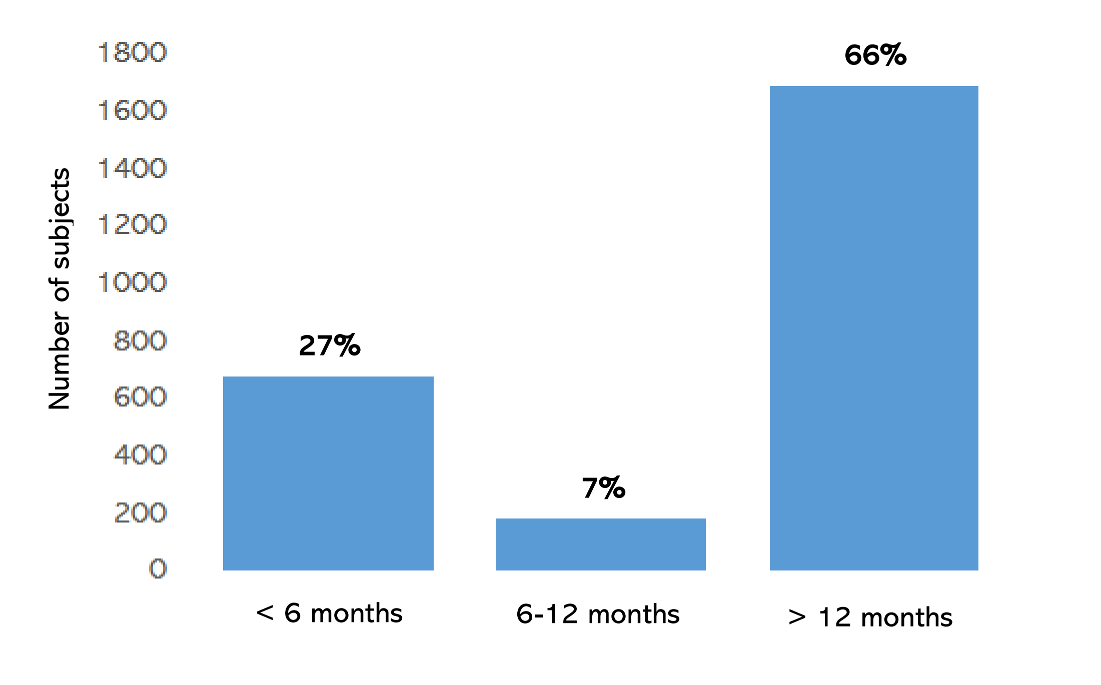

COPD: chronic obstructive pulmonary disease, N: number of subjects, SD: standard deviation

Supplementary Table 3

(a) Subject allocation based on gender and ABCD assessment

		MALE	FEMALE
ABCD classification	A	483 (19%)	151 (16%)
	B	1525 (59%)	539 (59%)
	C	59 (2%)	20 (2%)
	D	523 (20%)	209 (23%)
	TOTAL	2590	919

(b) Subject allocation based on body mass index and ABCD assessment

		< 25	25 – 29.9	> 30
ABCD classification	A	169 (17%)	281 (19%)	185 (18%)
	B	556 (57%)	895 (59%)	620 (60%)
	C	21 (2%)	33 (2%)	25 (2%)
	D	229 (24%)	299 (20%)	206 (20%)
	TOTAL	975	1508	1036

Supplementary Table 4

List of the comorbid conditions present in COPD patients

COMORBID CONDITIONS		CASES
Cardiovascular disorders	Heart failure, ischemic heart disease, arrhythmias, peripheral vascular disease, hypertension, stroke, carotid artery atheromatosis, valve dysfunction, ocular artery thrombosis, pulmonary embolism, pulmonary hypertension	2707 (59.6%)
Endocrine and metabolic disorders	Diabetes mellitus & metabolic syndrome, dyslipidemia, obesity, thyroid disease, thyroidectomy, hyperuricemia	684 (15.1%)
Neuropsychiatric problems	Anxiety, depression, bipolar disease, epilepsy, multiple sclerosis, Parkinson's disease, vertigo, dementia, trigeminal neuralgia, schizophrenia	478 (10.5%)
Gastroenterological problems	Gastroesophageal reflux, alcoholic hepatitis/ steatohepatitis, gastrectomy, bowel obstruction, colon diverticulum, ulcers, umbilical hernia, irritable bowel syndrome, choledocholithiasis, chronic constipation	259 (5.7%)
Oncological cases	Solid or hematological, benign or malignant	173 (3.8%)
Myoskeletal problems	Osteoporosis, osteoarthritis, bone fractures, spine stenosis	163 (3.6%)
Autoimmune disorders, chronic infections, and allergies	Allergy, hashimoto disease, hepatitis, tuberculosis, psoriasis, vasculitis, ankylosing spondylitis, colitis, rheumatoid arthritis, rheumatoid polymyalgia, lupus erythematosus, other	44 (0.9%)
Other problems	Other respiratory disorders, renal disorders, hematological disorders, or ophthalmological problems	36 (0.8%)
	TOTAL	4544 (100%)

COPD: chronic obstructive pulmonary disease

Supplementary Figure 4

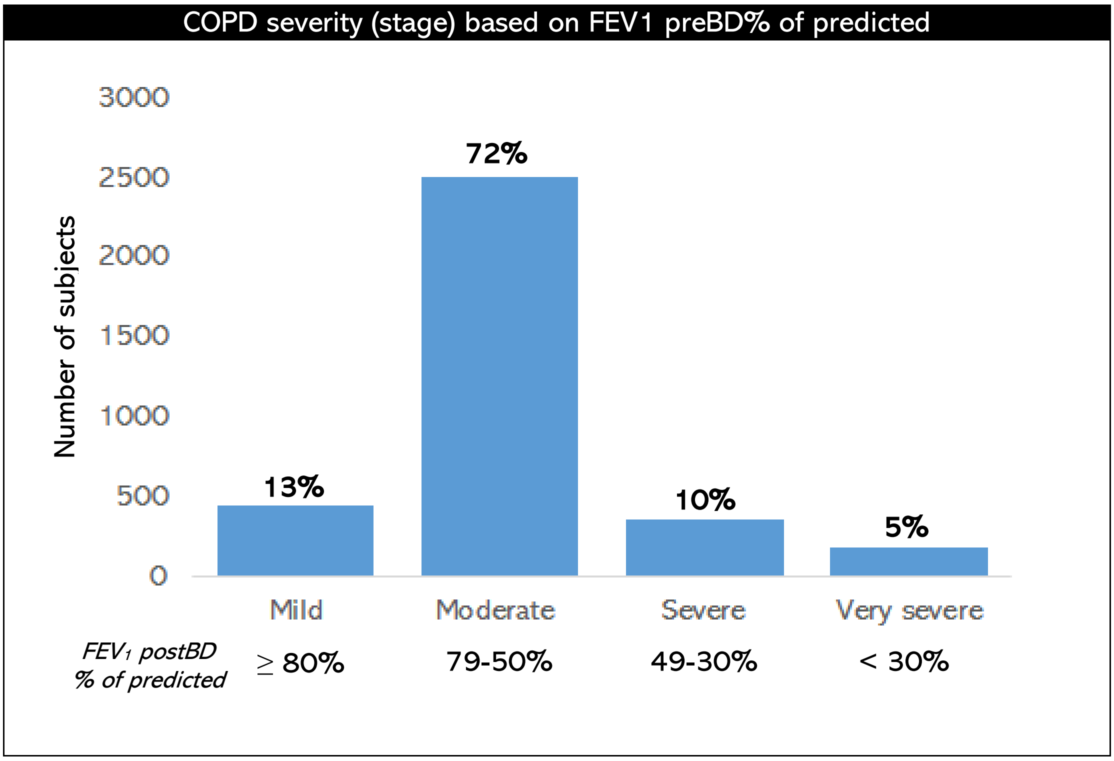

FEV_1: forced expiratory volume of the first second, FVC: forced vital capacity, postBD: post bronchodilation, postPD: post bronchodilation, SD: standard deviation

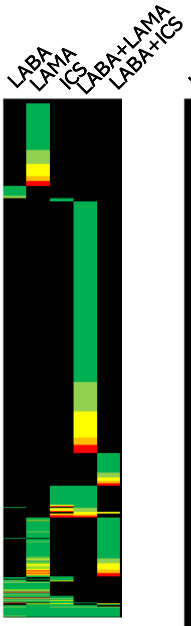

A

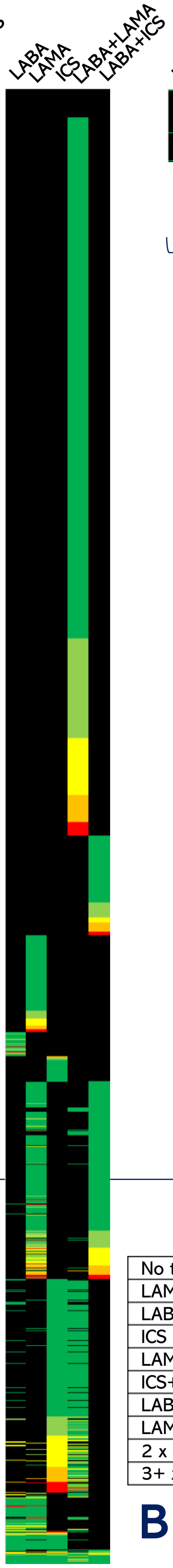

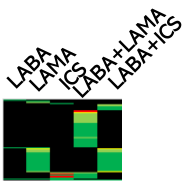

C

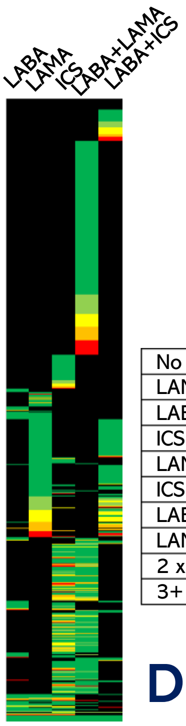

D

No treatment	13
LAMA	18
LABA	10
ICS	40
LAMA+LABA	261
ICS+LABA	53
LABA+LAMA+ICS	314
LAMA+ICS	7
2 x LAMA or LABA+ICS	2
3+ x bronchodilators (±ICS)	16

No treatment	0
LAMA	3
LABA	1
ICS	1
LAMA+LABA	37
ICS+LABA	6
LABA+LAMA+ICS	30
LAMA+ICS	0
2 x LAMA or LABA+ICS	0
3+ x bronchodilators (±ICS)	1

No treatment	6
LAMA	101
LABA	15
ICS	4
LAMA+LABA	326
ICS+LABA	44
LABA+LAMA+ICS	115
LAMA+ICS	2
2 x LAMA or LABA+ICS	1
3+ x bronchodilators (±ICS)	21

No treatment	40
LAMA	136
LABA	34
ICS	35
LAMA+LABA	1049
ICS+LABA	165
LABA+LAMA+ICS	521
LAMA+ICS	11
2 x LAMA/LABA+ICS	2
3+ x bronchodilators (±ICS)	79

B

No missing doses

Frequency of missing doses: 1/month

Frequency of missing doses: 2/month

Frequency of missing doses: 1/week

Frequency of missing doses: 2/week

No use of that drug

Supplementary Figure 5

Colourmaps depicting the combination of principal treatments (LAMA, LABA, ICS and their combination) used by the study participants per group of the ABCD assessment tool, and their compliance to each of them. Each colourmap consists of 5 columns, one per treatment type, and multiple compressed rows, one per subject (635 for group A, 2072 for group B, 79 for group C and 734 for group D). Black cells indicate that the corresponding treatment type is not used by the corresponding subject. Non-black cells indicate that the corresponding treatment type is used by the corresponding subject; colour differences (dark green, light green, yellow, orange and red) indicate differences in the consistency by which subjects receive the doses of the corresponding treatment types on a weekly or monthly basis. The accompanying small tables show the number of the different treatment strategies prescribed to the subjects per ABCD group.

ICS: inhaled corticosteroids, LABA: long-acting β adrenoreceptor agonists, LAMA: long-acting muscarinic receptor antagonists

Supplementary Table 5

Use of other pharmacological agents, for symptomatic treatment, besides the main treatment options for the chronic management of COPD (LAMA, LABA, ICS)

PHARMACOLOGICAL AGENT CATEGORY	USED	NOT USED
SABA	553 (15.5%)	3009 (84.5%)
SAMA	169 (4.7%)	3389 (95.3%)
Methylxanthines	118 (3.3%)	3434 (96.7%)
Oral glucocorticoids	58 (1.6%)	3495 (98.4%)
PDE4 inhibitors	169 (4.8%)	3388 (95.2%)
Antibiotics	110 (3.1%)	3442 (96.9%)
Antioxidants	50 (1.4%)	3501 (98.6%)
Mucolytic agents	272 (7.7%)	3282 (92.3%)

ICS: inhaled corticosteroids, LABA: long-acting β adrenoreceptor agonists, LAMA: long-acting muscarinic receptor antagonists, SABA: short-acting β adrenoreceptor agonists, SAMA: short-acting muscarinic receptor antagonists

Supplementary Table 6

Distribution of vaccinated patients among ABCD groups

	No vaccination	Influenza	S. pneumoniae	Both	TOTAL
A	137 (21.9%)	57 (9.1%)	83 (13.2%)	350 (55.8%)	627
B	392 (19.1%)	182 (8.9%)	242 (11.8%)	1232 (60.2%)	2048
C	22 (28.2%)	4 (5.15)	12 (15.4%)	40 (51.3%)	78
D	109 (15%)	59 (8.1%)	89 (12.2%)	471 (64.7)	728

Supplementary Table 7

Compliance of the study sample to the different COPD-related treatment categories

Chronic COPD treatment (main treatment options)	%COMPLIANCE		
	Perfect	Good	Poor
LAMA	71.5%	24.5%	4%
LABA	74.6%	21.7%	3.7%
ICS	66.7%	27.7%	5.6%
LAMA+LABA	74.6%	22.8%	2.6%
ICS+LABA	69.9%	26.1%	4%
Symptomatic relief (supporting treatment options)	Perfect	Good	Poor
SABA	64.6%	22.8%	12.6%
SAMA	67.9%	22%	10.1%
Methylxanthines	68.6%	23.6%	7.8%
Oral glucocorticoids	76.4%	21.8%	1.8%
PDE4 inhibitors	70.4%	27.1%	2.5%
Antibiotics	84.5%	13.7%	1.8%
Antioxidants	51.1%	37.8%	11.1%
Mucolytic agents	51.5%	34.3%	14.2%

%Compliance: the percentage of patients using each treatment category, who show either a perfect or a good or a poor compliance to taking the corresponding drug

Perfect compliance: the patient misses no doses monthly, Good compliance: patient misses one dose weekly at the worst case scenario, Poor compliance: patient misses two or more doses weekly

ICS: inhaled corticosteroids, LABA: long-acting β adrenoreceptor agonists, LAMA: long-acting muscarinic receptor antagonists, SABA: short-acting β adrenoreceptor agonists, SAMA: short-acting muscarinic receptor antagonists
